# A New Scientific Medical Model: Improvement of the Current Biopsychosocial Medical Models

**DOI:** 10.34133/research.0541

**Published:** 2024-12-03

**Authors:** Kezhen Zhang

**Affiliations:** ^1^ Taijitang Traditional Medicine Hospital, Beijing, China.; ^2^ Shandong University of Traditional Medicine, Shandong, China.

## Abstract

The current biopsychosocial medical models have substantial limitations and require to form an improved scientific medical model. This new model should consider human health holistically, emphasizing the integrity of life and focusing on the impact of physiological spaces, natural factors, and the interaction between individuals and their environment. We propose a “life–society–nature medical model” that provides novel perspectives for innovation in basic medical theory, the integration of Traditional Chinese and Western medicine, disease diagnosis, therapy and prevention, as well as the development of new therapeutic agents, scientific instruments, and approaches to medical education.

## Analysis of Current Medical Models and Their Deficiencies

Medical models are the fundamental ideas and primary methods for observing and dealing with medical issues that have gradually formed during the process of human medical exploration and practice. They are a high-level generalization of the overall understanding of life, health, and disease, functioning as the guiding principles and starting points that people adhere to when researching medical problems.

Medical models evolve correspondingly with societal progress. These changes align with human philosophical thinking, social transformations, scientific and technological advancements, and new understandings of the world, life, disease, etc. During the primitive era of human history, ever since the earliest theistic medical model, natural philosophy, mechanistic, and biomedical models had been successively experienced.

In 1977, George L. Engel, a professor of psychiatry and medicine in the Medical School at the University of Rochester, published an article titled “The need for a new medical model: A challenge for biomedicine” in the journal *Science* [1], putting forward the “biopsychosocial medical model”. However, in view of the numerous blind spots in medical theory and the many clinically encountered diseases with unknown etiology that are challenging to cure, this medical model still has its limitations.

To resolve medical problems, we need to clarify at least the following 3 fundamental questions:

First, what is the human body composed of?

Second, what are the causes of human diseases?

Third, how should we properly treat diseases when they occur?

The current medical model cannot adequately answer the above 3 questions, resulting in the following deficiencies. First, there are obvious deficiencies in the understanding of the structural elements of the human body in this medical model. In our previous studies of the human body, we primarily interpreted and analyzed the human body structure with a focus on physical entities while neglecting another widely existing component of the human body, that is, the physiological spaces within the body. Between organs, between tissues, and within organs and tissues, spaces are ubiquitous (see Figure). The human body is composed of both spaces and entities, and physiological spaces play a decisive role in metabolic processes [2]. For example, a lung is a hollow organ. In addition to the trachea, bronchi, alveoli, and other physical structures that we can observe through dissection or microscopy, there are also spaces. Without the spaces in the trachea and alveoli, the lung would lose their ability to facilitate gas exchange in the blood. The same holds true for the heart, blood vessels, intestines, etc.

**Figure.**
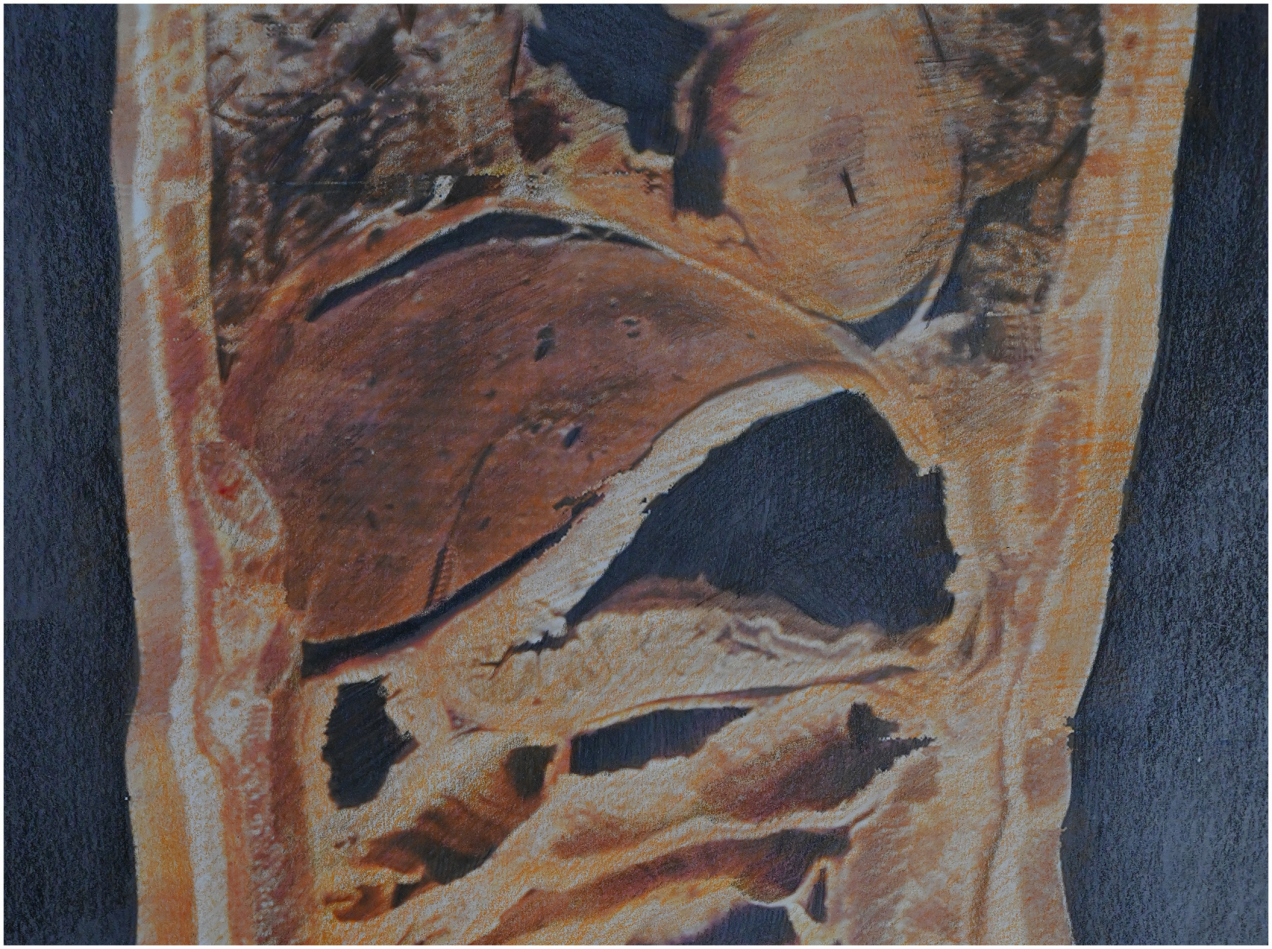
The organs, tissues, and interior of organs in the human body are filled with spaces [4].

Examining the physiological space, we can gain a more comprehensive understanding of the human body’s structures and the interrelationships. We fully grasp the intricacies of human anatomy and the complex interactions that underlie biological functions by understanding the physiological space in human body.

Second, there are apparent deficiencies in the understanding of the compositional elements of medical models in this model. The “biopsychosocial medical model” recognizes the impact of psychological and social factors on life and health but overlooks natural factors. The origin and growth of human life are inseparable from natural factors, and many human diseases are closely related to natural factors. For instance, when the temperature drops suddenly, many people will simultaneously “catch a cold”. This is precisely the impact of natural factors on human health. The decrease in temperature will also seriously affect human physiological functions, such as blood pressure, heart rate, respiration, and other indicators [3]. The lack of understanding of natural factors means that a series of medical theoretical issues and clinical practice problems (including understanding of etiology, disease diagnosis, therapy, and prevention) caused by natural factors have major defects.

Third, there are evident omissions in the understanding of disease etiology in this medical model. If life is studied and analyzed as a complex system, problems in the following 3 aspects may lead to the occurrence of diseases.

The first aspect is abnormalities in the elements that constitute life. If the elements that make up life become abnormal, it will inevitably lead to the occurrence of diseases. For example, if the organs, tissues, and cells that we currently recognize as visible to the naked eye become abnormal, it may lead to the occurrence of diseases.

The second aspect is abnormalities in the factors that affect life. If the human body itself has difficulty adapting to factors that affect life, it will also lead to the occurrence of diseases. For instance, overeating leads to indigestion, and low temperatures lead to frostbite.

The third aspect is abnormalities in the interrelationships between various elements in the process of life metabolism. Abnormalities in the interrelationships between the constituent elements of life, or between the constituent elements and influencing factors, will also lead to the occurrence of diseases. For example, patients can feel pain but cannot find a physical cause through laboratory tests. Such diseases are very likely caused by abnormalities in the interrelationships between various elements. The diseases are closely relevant to whether the physiological space in the human body is functional normally. Human being’s physiological space is the prerequisite for the existence of organs, tissues, and cells, the place where functions are realized, the channels for the supply of required energy, and the premise for the realization of the interrelationships between various metabolic elements in the human body.

Fourth, there are obvious deficiencies in the interpretation of the relationship between psychology and physical structure in the existing medical model. The “biopsychosocial medical model” makes us aware of the pathogenic effects of psychological factors. However, psychological factors do not exist in isolation from the body, but are closely related to physical structures, interacting and influencing each other. Psychological changes can cause obvious physiological changes in the body, and physiological changes can also cause changes in psychological emotions. For example, during special periods such as menopause, menstruation, and puberty, emotional changes are notably different from usual.

Fifth, there are obvious deficiencies in the medical theory guided by this medical model in terms of clinical treatment and prevention of diseases. Currently, in clinical treatment, we often target the symptoms of the disease, the patient’s signs, and the abnormal indicators obtained through laboratory tests, and these 3 aspects are 3 different types of results produced after the cause of the disease acts on the body. Result-oriented therapy that ignores the cause not only cannot truly cure the disease, but the side effects of drugs will cause new problems.

## Requirements for Constructing a New Medical Model

To overcome the deficiencies in the current medical model, we need to construct a more complete medical model.

First, the new medical model must withstand scrutiny at a philosophical level. As a discipline that investigates the essence of the fundamental and universal aspects of the world, philosophy provides a foundation upon which a sound medical model should be built, conforming to its basic principles. For instance, the relationships between “existence and emptiness” and “cause and effect” are essential philosophical concepts. In the context of medicine, it is crucial to elucidate how diseases manifest from nonexistence to existence and to identify the causes underlying various diseases.

Second, the new medical model must meet the development requirements of modern scientific theories. The development of medical models is dependent on scientific thought innovation and scientific and technological progress. For example, the “biopsychosocial medical model” proposed by G. L. Engel was based on the organic concept in biology proposed in Bertalanffy’s “general system theory”, emphasizing considering organisms as a whole or system.

It is evident that the development of medical models also needs to be supplemented and improved by the latest scientific theoretical achievements in a timely manner. Currently, big data, artificial intelligence technology, and smart wearable devices provide us with more perspectives and data support for studying diseases. They can sense even more intuitively the objective existence of human body spaces within microscopic structures and the impact of psychological and natural factors on the body.

Third, the new medical model must play a guiding role in basic medical theories. The new medical model must overcome the inadequacies in understanding the following 3 aspects in previous medical models: insufficient understanding of life’s structure and its operational laws, lack of understanding in etiology, and deficiencies in the guiding methods or directions of medical practices.

Fourth, the new medical model must play a guiding role in medical practice. Currently, there are many difficult and unknown diseases or disease phenomena in medicine. Many diseases cannot be truly effectively treated in practice. The new medical model must be able to guide and solve such problems.

Fifth, the new medical model must be logically self-consistent. It is crucial that a novel medical model ensures cognitive consistency and logical coherence. The model should possess the capability to systematically interpret, theoretically support, predict, and guide practice for previously unknown phenomena that emerge in theoretical research and clinical practice.

## The Construction of a New Medical Model

The new medical model should scientifically interpret life, effectively address the relationship between humans and nature, and elucidate the various factors that influence the onset and progression of disease.

First, life is a complete, organic whole system. The human psyche and body are an inseparable entity. When the physical structure interacts with the external environment, it gives rise to corresponding expressions of psychological activity. The new medical model must incorporate the concept of humans as complete, organic beings into the research framework of medicine. In the treatment of certain emotional disorders, attention should be given not only to the patient’s psychological states but also to their physical conditions, as the 2 are interconnected and mutually influential.

Second, the human body is a complex open system. In the new medical model, it should be recognized that physiological space is an essential part of the human body structure. Space is a prerequisite for the realization of the functions of various constituent elements of the human body itself and a prerequisite for the realization of the interrelationships between various elements.

Each individual lives within a social and natural environment. Therefore, our health is determined by multiple factors, including our physical and mental well-being, the energy required by the body, and the dynamic processes within the body. In addition, the state of our health is influenced by numerous external factors, such as society and nature. These complex interrelationships far exceed the scope of individual life and current medical understanding. However, life is constantly affected by these factors, which are also important causes of diseases. In the process of these mutual influences and interrelationships, the physiological space within the human body plays a decisive role. The interactions between the internal elements of the human body and the interactions between the internal and external elements can be truly realized through the normal physiological space within the human body. Otherwise, once the coordination between them is disrupted, it will result in the occurrence of diseases.

Third, man and nature are an integral whole. Incorporating natural factors into the new medical model is of great significance for our correct understanding of life structure, disease causes, disease diagnosis, therapy, and prevention.

Regrettably, natural factors are not included in the existing medical model, which only includes biological, psychological, and social factors. However, the impact of natural factors on life is more extensive, universal, primitive, and long-standing. Nature is the soil for the origin and evolution of human beings and the foundation of human survival. Any changes in nature will affect the state of human life. Since we have not truly recognized the pathogenic effects of natural factors such as wind, cold, and dampness, we cannot correctly interpret such diseases from a theoretical perspective, nor can we find the correct methods for diagnosis, therapy, and prevention in clinical practice. We can only seek causes and guide clinical practice based on existing biological and psychological factors.

Therefore, considering the deficiencies in the current “biopsychosocial medical model” and drawing upon the experience gained from theoretical innovation research and clinical practice, we propose the establishment of a more comprehensive new medical model—the “life–society–nature medical model”. This new medical model provides us with a comprehensive and systematic framework for understanding and exploring various complex and unknown disease phenomena. It emphasizes that the elements that constitute life, the elements that influence life, and the interrelationships between these elements can all be potential causes of various diseases. The establishment of this medical model has important implications for future medical theoretical innovation and clinical practice.

## The Significance of the New Medical Model

First, the establishment of a new medical model changes the original medical viewpoint. Life is not a simple, reducible, tangible structure, but an organic whole with flesh and blood, psychological conscious activities, and openness.

Second, the new medical model incorporates natural factors into the medical model, which not only means the integrity of man, society, and nature, but, more importantly, we need to pay attention to the impact of natural factors on life and health.

Third, theoretical problems that cannot be interpreted by the original medical model can obtain new solutions under the guidance of the new medical model.

Fourth, the new medical model can provide safe and effective therapy strategies and methods for challenging diseases. 

Fifth, the new medical model has crucial implications for guiding the future development of medicine. This guiding influence extends to various domains, including innovation in basic medical theory, the integration of traditional Chinese medicine and Western medicine, disease diagnosis, therapy and prevention, the development of novel pharmaceuticals and scientific instruments, medical education, and other related fields.
